# The Potential of Nanopore Technologies in Peptide and Protein Sensing for Biomarker Detection

**DOI:** 10.3390/bios15080540

**Published:** 2025-08-16

**Authors:** Iuliana Șoldănescu, Andrei Lobiuc, Olga Adriana Caliman-Sturdza, Mihai Covasa, Serghei Mangul, Mihai Dimian

**Affiliations:** 1 Integrated Center for Research, Development, and Innovation for Advanced Materials, Nanotechnologies, Manufacturing and Control Distributed Systems (MANSiD), Stefan cel Mare University of Suceava, 720229 Suceava, Romania; iuliana.soldanescu@usm.ro (I.Ș.); dimian@usm.ro (M.D.); 2 Department of Computer, Electronics and Automation, Stefan cel Mare University of Suceava, 720229 Suceava, Romania; 3 Department of Biological and Morphological Sciences, College of Medicine and Biological Sciences, Stefan cel Mare University of Suceava, 720229 Suceava, Romania; mcovasa@usm.ro (M.C.); serghei.mangul@gmail.com (S.M.); 4 Department of Clinical and Surgical Sciences, College of Medicine and Biological Sciences, Stefan cel Mare University of Suceava, 720229 Suceava, Romania; 5 Department of Computers, Informatics, and Microelectronics, Technical University of Moldova, 2045 Chisinau, Moldova; 6 Department of Clinical Pharmacy, Alfred E. Mann School of Pharmacy and Pharmaceutical Sciences, University of Southern California, Los Angeles, CA 90089, USA

**Keywords:** proteomics, amino acids, bioinformatics, personalized medicine, nanotechnology

## Abstract

The increasing demand for high-throughput, real-time, and single-molecule protein analysis in precision medicine has propelled the development of novel sensing technologies. Among these, nanopore-based methods have garnered significant attention for their unique capabilities, including label-free detection, ultra-sensitivity, and the potential for miniaturization and portability. Originally designed for nucleic acid sequencing, nanopore technology is now being adapted for peptide and protein analysis, offering promising applications in biomarker discovery and disease diagnostics. This review examines the latest advances in biological, solid-state, and hybrid nanopores for protein sensing, focusing on their ability to detect amino acid sequences, structural variants, post-translational modifications, and dynamic protein–protein or protein–drug interactions. We critically compare these systems to conventional proteomic techniques, such as mass spectrometry and immunoassays, discussing advantages and persistent technical challenges, including translocation control and signal deconvolution. Particular emphasis is placed on recent advances in protein sequencing using biological and solid-state nanopores and the integration of machine learning and signal-processing algorithms that enhance the resolution and accuracy of protein identification. Nanopore protein sensing represents a disruptive innovation in biosensing, with the potential to revolutionize clinical diagnostics, therapeutic monitoring, and personalized healthcare.

## 1. Introduction

Peptides and proteins constitute the structural and functional foundations of all living organisms, from simple viruses to complex human systems. Starting with genetic information encoded in nucleic acids, the pathways leading to protein synthesis are intricate, allowing for extensive functionalization and diversification of proteins but also presenting multiple points where errors may arise [1]. Protein structure or concentration variations can profoundly impact biological processes [2,3], as they influence protein abundance, functionality, and interactions within the organism [2]. For instance, alterations in protein degradation rates, cellular distribution, or enzyme kinetics can disrupt physiological balance, potentially leading to disease states [4]. This highlights the importance of accurately identifying and quantifying proteins across various biological conditions. However, a significant challenge in protein analysis, especially through sequencing, lies in interpreting results when structurally similar proteins are present. Such similarities can introduce substantial analytical errors, as more than 15% of human proteins may contain at least one mistranslated amino acid, complicating accurate identification [2,5,6]. Each molecular variation introduces additional variability, necessitating rigorous analytical approaches to yield precise data and foster a clearer understanding of protein function and pathology. To address these complexities, advanced analytical methods are essential to differentiate between closely related protein variants effectively [7].

A critical evaluation of the classical methods and the nanopore method reveals that the latter has distinct advantages in real-time single-molecule analysis. The nanopore method has been demonstrated to facilitate the identification of amino acid sequences and post-translational modifications [8]. Moreover, due to their capacity to discern disparities at the molecular level, nanopores have emerged as a significant instrument in the identification of novel biomarkers and personalized clinical diagnostics, thereby providing novel insights into the structure, functions, and interactions of proteins in biological and pathological contexts [9]. Furthermore, the nanopore technique is characterized by its portability, thus classifying it as a point-of-care diagnostic technique [10]. Despite these promising advances, several technical challenges remain, such as controlling the translocation speed of proteins through nanopores, protein unfolding, and managing the signal-to-noise ratio, which remain active areas of research and optimization [11].

## 2. Biomarkers: Identification and Detection Methods

Recognizing the need to identify specific biomarkers for a wide array of diseases is a fundamental driver of research. Advances in molecular analysis technologies have significantly enhanced diagnostic accuracy and the effectiveness of personalized medicine, leading to remarkable outcomes [12]. The discovery of novel peptide biomarkers has the potential to greatly influence disease progression, management, and intervention strategies [13]. Biomarkers play a crucial role in early disease detection, accurate diagnosis, tailored treatment planning, monitoring therapeutic responses, and drug development [14]. The significance of proteins as biomarkers has been well established [15], from insulin, a critical marker in diabetes management, to proteins with mechanisms of action still under investigation, such as beta-amyloid in Alzheimer’s disease [16]. Proteins are also key in rare diseases, like Huntington’s disease (HD), where abnormal mutations in the huntingtin protein drive neurodegeneration [17]. Understanding the role of proteins in physiological processes provides invaluable insights into an individual’s health status. This requires analyzing protein levels in biological samples and uncovering their mechanisms of action, necessitating complex quantitative and qualitative analyses.

Comprehensive monitoring of protein levels in biological samples (e.g., blood, urine, and cerebrospinal fluid) is essential for diagnosing and managing diseases, predicting disease risk, and evaluating therapeutic responses. Protein levels can fluctuate significantly under certain conditions, with C-reactive protein (CRP) being a well-known example; CRP levels rise substantially during inflammatory processes [15]. Some proteins serve as disease-specific biomarkers, such as prostate-specific antigen (PSA), where elevated levels raise suspicion of prostate cancer [18]. The number of diseases in which proteins serve as key biomarkers continues to grow, highlighting the need for precise quantitative and qualitative biomarker analyses. Moreover, proteins play a critical role in targeted therapies and personalized medicine, providing insight into disease mechanisms and therapeutic responses. Quantitative protein analysis is frequently used in routine diagnostics to assess health status and potentially predict physiological disturbances [7]. A major challenge is identifying post-translational modifications and other molecular changes that reveal cellular-level processes and functions, offering a deeper understanding of disease pathways and potential interventions [19,20].

Currently, mass spectrometry is the most widely used method for analyzing novel protein biomarkers due to its ability to handle complex samples and identify post-translational modifications [21,22]. Another method, Edman degradation, selectively cleaves the N-terminal amino acid without damaging the rest of the peptide chain, allowing for sequential amino acid identification [23]. While mass spectrometry is favored in modern proteomics for its versatility, Edman degradation is limited to determining the sequence of N-terminal amino acids only [24]. Both techniques involve complex sample preparation, including extraction, purification, and enzymatic digestion, which require specialized expertise. Steps such as ultrafiltration or solid-phase extraction are necessary to isolate proteins from other sample components. Additionally, proteins often adhere to equipment surfaces (e.g., chromatography tubing), which can lead to sample loss. Mass spectrometry systems are highly costly, often reaching hundreds of thousands of dollars [21], while Edman degradation setups are more affordable but demand precise laboratory conditions, as the reactions are sensitive to temperature and humidity [25,26,27]. The utilization of mass spectrometry has become increasingly prevalent due to its ease of use and substantial throughput capabilities. In contrast, the application of Edman degradation has seen a decline in recent years.

In clinical laboratories, immunoassays are commonly used for protein detection but are only feasible when the protein of interest is known [28]. This technique requires specific antibodies that exclusively bind to the target protein, forming an antigen–antibody complex that can then be measured via the immunoassay [29]. A wide range of methods are available in the literature for both qualitative and quantitative protein analysis, depending on the type of protein, the purpose of the analysis, and available resources. Research laboratories typically focus on understanding protein functions and properties, while accredited clinical laboratories prioritize diagnosis and patient monitoring. Table 1 summarizes current methods for quantitative and qualitative protein analysis, highlighting the principle of each method and its main advantages and limitations. As separation is an important step in protein analysis, protein separation methods such as electrophoresis are also considered. This highlights the need for continued development of techniques to overcome existing challenges in protein analysis, ultimately facilitating accurate and comprehensive data collection that advances the life sciences.

The proteomic approach not only aids in disease diagnosis but also enhances the prediction of treatment responses and enables the monitoring of disease progression. Additionally, analyzing proteins involved in various diseases offers insights into underlying disease mechanisms, facilitating the development of targeted therapies [47]. The nanopore technique for protein analysis is currently under development, being used only in laboratory research and not integrated into clinical trials. However, it is important to highlight the evolution of the method given that it presents distinct advantages over traditional methods, including real-time monitoring, high sensitivity, and superior resolution. Given the diverse roles of proteins in numerous pathologies, it is crucial to understand their impact on disease mechanisms, which can lead to significant advancements in both diagnostics and therapeutics.

## 3. Clinical Utility and Translational Potential

Nanopore technology has demonstrated significant clinical utility in cardiovascular diagnostics. The detection of natriuretic peptides (ANP, BNP, and CNP)—key biomarkers for heart failure—has been successfully achieved with diagnostic sensitivity meeting European Society of Cardiology guidelines [48]. The technology’s ability to discriminate between different peptide forms and detect post-translational modifications provides enhanced diagnostic accuracy compared to conventional immunoassays.

Also, multiple studies have validated nanopore technology for cancer biomarker detection across various malignancies. The platform has successfully detected prostate-specific antigen (PSA) for prostate cancer screening [49], carcinoembryonic antigen (CEA) for colorectal cancer monitoring, and human epididymis protein 4 (HE4) for ovarian cancer diagnosis [50]. The technology’s ability to detect protein modifications and degradation products provides additional diagnostic information not readily available through traditional methods.

Nanopore-based pathogen detection represents a rapidly growing application area, particularly highlighted during the COVID-19 pandemic [9]. The technology enables direct detection of pathogen-derived proteins and peptides from clinical samples, offering advantages in speed and sensitivity over traditional culture-based methods. The ability to detect multiple pathogens simultaneously makes nanopore technology particularly valuable for syndromic testing approaches.

The miniaturization potential of nanopore technology makes it particularly suitable for point-of-care applications [51,52]. Portable nanopore devices have been developed that can perform protein biomarker detection in resource-limited settings, addressing critical healthcare access issues. The combination of rapid results, minimal sample preparation, and instrument portability positions nanopore technology as a transformative point-of-care diagnostic platform.

## 4. Nanopore Setups and Applications for Protein and Peptide Detection

### 4.1. Types of Nanopores and Their Limitations

**Figure 1:**
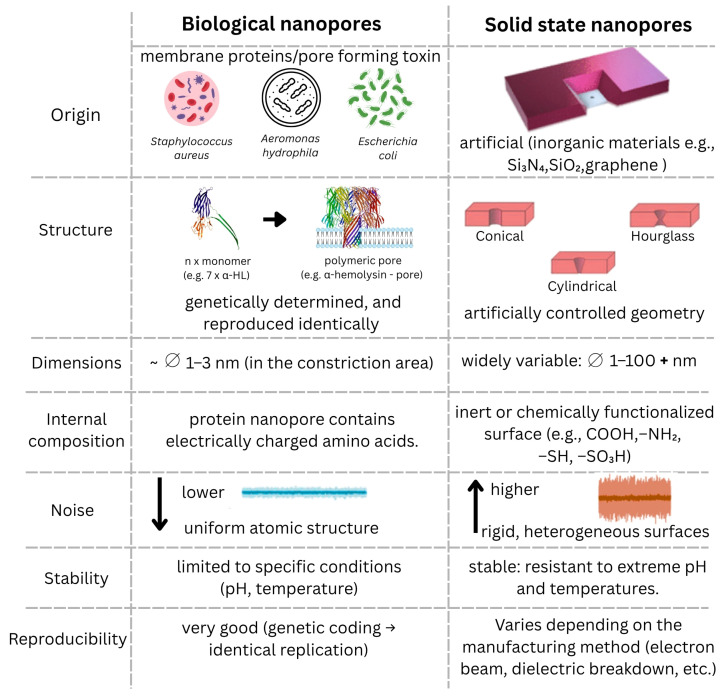
A comparison of biological and solid nanopores highlights essential differences in their origin, structure, dimensions, internal composition, and functional characteristics. (arrows indicate increase or, respectively, decrease in noise amplitude; Ø represents diameter of the nanopore).

Following the discovery of natural protein nanopores like α-hemolysin (α-HL) [53,54,55,56,57] researchers sought ways to modify these structures to suit experimental needs and better match the properties of target molecules. This led to the concept of fabricating nanopores from various materials, such as silicon nitride, silicon oxide, glass, and graphene, often using focused electron beam techniques to create nanopores on the nanometer scale [58]. Figure 1 provides a visual comparison between biological and solid nanopores, highlighting the essential differences related to the origin, structure, pore size, internal composition, background noise, stability, and reproducibility of each type. Knowledge of these differences is essential for the appropriate choice of nanopore type, depending on the specificity of the molecule being analyzed, the experimental conditions, and the purpose of the application.

**Figure 2:**
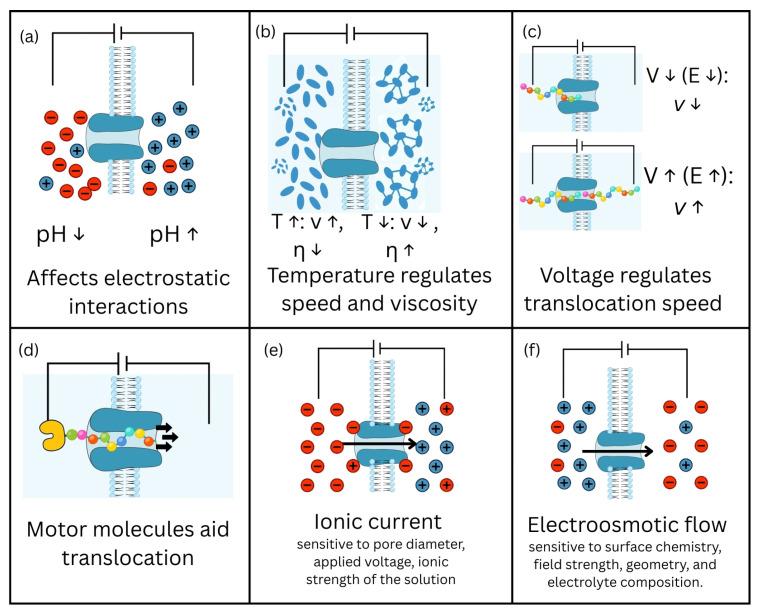
Factors that influence the transport of molecules through nanopores. Main experimental conditions influencing the processes of molecule translocation through nanopores. (**a**) pH influences the concentration of H^+^ and OH^−^ ions, determining the acidic (pH < 7), neutral (pH = 7), or basic (pH > 7) nature of the medium, which changes the electric charge of molecules and their interactions with the pore walls. (**b**) Temperature affects viscosity (η) and speed (v), such that a decrease in temperature causes an increase in viscosity and a reduction in molecular mobility, while an increase in temperature has the opposite effect. (**c**) The applied voltage (V) determines the intensity of the electric field (E) and the speed (v) of the molecules. (**d**) Motor proteins may be involved in the active, directed translocation of molecules, contributing to the control of the speed and direction of movement. (**e**) Ionic current represents the flow of ions determined by the potential difference applied to the ends of the pore. (**f**) Electroosmotic flow describes the movement of fluid through the pore (arrows indicate increases or, respectively, decreases in values of pH, temperature, viscosity, speed, voltage, electrical field, respectively; “-“ and “+” represent electrical charge).

Currently, it is challenging to definitively determine which type of nanopore is optimal, as their efficiency depends on multiple factors beyond the material itself. As shown in Figure 2, the translocation of a molecule depends on various factors, the physicochemical processes inside it.

In certain protein detection methods, molecules do not completely cross the pore but are captured or retained at the entrance, causing significant current blockages without effective translocation [57,58,59]. Both biological and solid nanopores require specific adjustments, such as the addition of motor molecules or the tuning of the electrical potential [60,61]. The effectiveness of nanopores is influenced by the electrophysiological environment, the purity of reagents, and even external noise levels [62].

In solid-state nanopores, high-frequency noise and 1/f noise are more common and affect signal resolution and event detection. Factors such as dielectric fluctuations of the membrane, electrode quality, and ionic strength contribute to base current instability and event classification errors [63,64,65]. Building on the success of nanopore sequencers for nucleic acid analysis, researchers are now exploring nanopore sequencing for other molecules, including proteins. Nanopore technology provides a powerful, versatile approach to real-time monitoring of individual protein molecules [66].

To improve the functionality of the nanopores, research was made to combine the two types of nanopores, resulting in hybrid nanopores [67]. Hybrid nanopores integrate biological components and solid materials, thus combining the advantages of both types of structures. These nanopores offer enhanced mechanical stability and high functional compatibility, enabling the detection of individual molecules with increased selectivity.

Furthermore, the properties of nanopores influence the interactions between them and molecules, including surface potential, electric charge distribution, Van der Waals force, electrostatic interactions, and hydrogen bonds [68,69,70]. Table 2 presents a systematic comparison of the main types of nanopores, categorized by their origin and several aspects related to their geometry and potential interactions with other molecules.

Transport processes through nanopores are complex and depend on a number of factors, including the electric field, electrophoretic forces, electroosmotic flux, pH, temperature, nanopore diameter, surface charge, Debye length, etc. [71,72,73,74,75].

**Table 2:** Classification of nanopores according to their origin (biological/solid), highlighting their geometrical and physico-chemical properties [76].

	Nanopore	Geometry and Size	Distinct Functional Characteristics	Refs.
Biological	α-Hemolysin 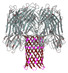	Mushroom-shaped, transmembrane heptamer. Outer protein vestibule and β-barrel in membrane Vestibule ~3.6 nm diameter and ~5 nm length; β-channel ~2.6 nm diameter and ~5 nm length Constriction zone~1.4 nm diameter	Robust and stable channel in lipid membranes Hydrogen bonds with polar residues inside the channel	[77,78,79]
	MspA (Mycobacterium smegmatis porin A) 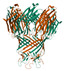	Conical shape (funnel shape)—symmetric octamer Diameter of ∼1.2 nm and a length of ∼0.6 nm	Short and very narrow channel (~2–4 residues occupy the narrowest area) leading to higher translocation rates and, potentially, higher resolution (fewer amino acids simultaneously in the pore)	[79,80]
	Aerolysin 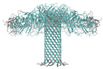	β-barrel Diameter ~1.0 nm, length ~10 nm	The lumen of the pore contains a high density of charged residues, which confer a pronounced electrostatic character to the interior. Very long and narrow pore 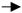 slow translocation and long-lasting signals	[81,82]
	ClyA (Cytolysin A) 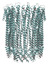	Barrel-shaped Diameter of cis vestibulum ~6 nm; diameter of trans vestibulum ~3 nm; total length ~13 nm	Strong electrostatic interactions with positively charged molecules; hydrogen bonds with polar residues. Large lumen that accepts native folded proteins	[83,84]
	OmpG (Outer membrane protein G) 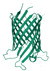	Monomer β-barrel with 14 chains Diameter ~2–2.9 nm; length ~3 nm	Naturally exhibits gating behavior due to the flexibility of the L6 loop, which can spontaneously block or unblock the channel	[85]
Solid-state	SiN 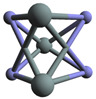	Cylindrical, thickness is flexible and can be reduced to < 5 nm Pore diameter can be controlled in manufacturing	Robust and chemically inert solid nanopore, compatible with CMOS processes; provides durability and precise pore size control	[79,86]
	Graphene 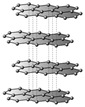	Two-dimensional sheet of atomic carbon (atomic thickness ~0.34 nm) Diameter~2 nm to 25 nm	Very ‘sticky’ hydrophobic surface, which can adsorb biopolymers and slow translocation	[87]
	MoS_2_ (Molybdenum disulphide) 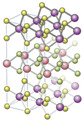	Membrane—monolayer of MoS_2_ ~0.7 nm thick Pore size has been observed to range from 1 to 2 nm	Detect molecular translocation via ionic signal (as a biological nanopore) Electrically amplifier response (via electronic effects, as a sensor)	[88]
Hybrid	α-Hemolysin in solid pore 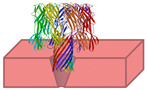	α-hemolysin protein pore (heptamer ~10 nm total diameter) inserted into a larger solid membrane nanopore (e.g., ~20 nm in SiN)	The biological pore controls interactions with translocated molecules, but the system becomes more mechanically stable due to the solid support	[89]
	Solid nanopores (SiN) functionalized with biomolecular ligand 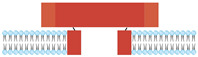	The basic geometry is still that of the solid pore. The functionalized molecule may reduce the effective pore size (e.g., a 10 nm diameter SiN pore with a DNA aptamer attached may have a smaller functional area)	Specific interactions are brought about by the attached biomolecular ligand, in addition to non-specific electrostatic interactions with the solid pore walls	[90]
	DNA Origami Nanopores 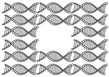	Customizable 2D and 3D structures Diameter between 2 and 20 nm	Charge distribution can be modified by nanopore design	[91,92,93,94]

The primary challenge in protein sequencing lies in ensuring that the protein translocates through the nanopore slowly, ideally one amino acid at a time, with a sufficiently long effective translocation time through the pore (e.g., ≥10 ms) so that the current signal can be distinguished for each amino acid. Even if translocation is sequential, too high a speed can lead to signal overlap and compromise the accuracy of identification [95,96]. Various strategies have been proposed in the literature to address this. One approach uses proteases to incrementally pull peptides through the nanopore [97]. Another involves stepwise control using a motor molecule, where a DNA–peptide complex is conjugated, and a helicase attached to the DNA drives the peptide through the nanopore, controlling its movement [98,99]. Despite significant advances in nanopore technology, especially in recent years, limitations such as low signal-to-noise ratio, difficulty in controlling the translocation rate, and challenges in differentiating structurally similar amino acids still require comparative studies between different experimental approaches [62,99,100].

### 4.2. Technological Considerations and Control Strategies for Protein Translocation

Nanopore technology has evolved in terms of strategies for analyzing proteins and amino acids at the single-molecule level. Before detailing specific technical specifications, it is important to contextualize the capabilities and limitations of nanopore techniques in comparison with established methods such as mass spectrometry.

Mass spectrometry is the standard in this field, being an integrated method in research, diagnostics, and industry. The nanopore method is a new technique at the research stage; however, it is believed to revolutionize molecular analysis. While mass spectrometry has reached a resolution of 300–500 amino acids for top-down proteomics [101], nanopore technologies promise to be able to read proteins of any length. Being at the research stage, nanopore technology encounters difficulties in translocation control, accuracy, noise, etc. However, due to its sensitivity at the single-molecule level, the nanopore method may be a valuable alternative to classical protein sequencing methods [102].

Numerous studies [103,104,105,106,107] have reported successfully achieving translocation, providing valuable insights into peptide concentration, peptide–peptide interactions, and amino acid sequences. Studying the status of research and the methods used to facilitate and optimize the translocation process is essential in understanding the mechanisms involved, identifying limiting factors, and developing effective strategies to improve performance.

#### 4.2.1. Challenges and Emerging Strategies in Protein Translocation and Unfolding

Several properties, including the translocation rate, the distribution of electric charge, surface interactions, and three-dimensional structures, amongst others, influence the process of protein translocation through nanopores. This section addresses the main obstacles associated with protein translocation and unfolding and reviews experimental progress.

One of the major challenges in nanopore-based protein analysis is unfolding the protein before entry, as folded proteins with complex three-dimensional structures often exceed the nanopore’s diameter, causing significant ion flow blockage. In the case of the α-HL pore, Congo Red (CR) has been used to facilitate the translocation of β-amyloid 42 molecules. CR appears to inhibit the aggregation of amyloid fibers, likely by weakening intermolecular bonding interactions, allowing the molecules to pass through the nanopore [108,109]. In an aerolysin nanopore, peptide translocation should produce distinct current blockages to enable the identification of the amino acid sequence. Attaching amino acids to a carrier, such as a heptapeptide arginine, slows the translocation rate, making the sequence easier to observe. By correlating blockage levels, as indicated by changes in current intensity, with the excluded volume or molecular mass of each amino acid, it has been observed that amino acids with higher molecular mass induce greater current blockages. The lower translocation velocity is beneficial in the context of nanopore analysis, improving temporal resolution. In the context of the aerolysin pore, the attachment of negatively charged amino acids to the positively charged carrier (heptapeptide arginine) reduces the positive charge and thus the electrophoretic force, which decreases the frequency of translocation events [110].

Preliminary studies focused on the enzymatic approach to protein unfolding. Using the biological nanopore α-HL enabled the translocation of specific peptides. Results demonstrated that molecular motors, such as ClpX, facilitate protein unfolding and translocation by utilizing ATP (adenosine triphosphate) hydrolysis. ClpX generates a pulling force of approximately 20 pN, allowing it to translocate up to 80 amino acids per second [111]. For these experiments, Smt3, a 98-amino acid protein containing both beta and alpha-helical regions, served as the test protein. Modifications were made to enhance and validate its translocation, including the attachment of negatively charged aspartate residues. The translocation events were detected by monitoring ionic current changes, which showed more than a 50% decrease as the peptide passed through the nanopore [112,113]. Recent advancements confirmed ClpX’s capability to translocate and sequentially “read” a folded protein using the MinION™ platform. The system utilizes the R9.4.1 cell that continues CsgG biological pores, the novelty of the method is the ClpX motor enzyme that facilitates the passage of the protein through the nanopores in 2 amino acid steps. The accuracy of the method is promising, in a variable region known to have 5 amino acid types (5-way classification), 86% of the identification was performed, and 28% for 20-way classification. In conditions where the readings are repeated for the same molecule, the accuracy can increase up to 61% for the identification of the 20 amino acids. The use of this enzyme is still in the research stage, as ClpX can successfully unfold tertiary protein structures but, depending on the physical and chemical properties of the peptide a slipping back of the enzyme can be observed [114].

In the case of unfolded proteins, a common challenge is the formation of ‘blob-like’ aggregates within the nanopore, which can obstruct translocation. To study and mitigate this issue, nanopores with varying inlet sizes and internal diameters were designed. Sauciuc et al. found that nanopores with smaller dimensions and non-adhesive inner surfaces facilitated smoother, linear transport of proteins, reducing the likelihood of blockages [115].

Comparisons with established methods, such as mass spectrometry, are essential for validating nanopore-based protein analysis. Studies have directly compared these approaches; for instance, a Fragaceatoxin C nanopore was used to identify individual proteins, with results subsequently compared to mass spectrometry analysis of the same proteins. The amplitude of the electric current generated during the translocation of a solid-state peptide was measured to estimate peptide size. Analysis of the Iex% (percentage excluded current) spectra demonstrated a strong correlation with mass spectrometry data, indicating that protein recognition may be achievable by comparing nanopore spectra with those from mass spectrometry [102].

#### 4.2.2. Analytical Strategies in Biological and Solid-State Nanopores

Biological and solid nanopores represent two distinct but complementary approaches to single-molecule protein analysis. This section presents relevant examples from the literature that highlight the applicability of each type of nanopore in protein analysis.

##### Protein Analysis with Biological Nanopores

Biological nanopores have been widely investigated for their specificity and structural uniformity. In one study [116], a motor enzyme was used with an MspA (Mycobacterium smegmatis porin A) [117] nanopore for DNA translocation, revealing that nucleotide passage occurs in 0.33 nm steps, which is comparable to the stepwise passage of amino acids. The structure of the DNA molecule also plays a significant role, as the DNA chain loads the motor enzyme, which drives the molecule through the pore, inducing changes in ionic current. Similarly, peptide translocation through nanopores produces stepwise changes in ionic current, with lower amplitude than DNA, as smaller amino acids create less obstruction in the nanopore than DNA nucleotides [99,116]. To validate this method, various peptide sequences were translocated through nanopores, and the differences between peptide variants were distinguished using a Markov model [118,119]. This approach yielded an identification accuracy exceeding 85%, a promising result compared to the initial accuracy achieved in early DNA sequencing experiments [99,120]. Given the technical challenges associated with protein analysis, these studies [99,116] have demonstrated that nanopore technology can be applied to protein sequencing with high resolution comparable to DNA sequencing.

The identification of all 20 amino acids using nanopore technology is progressing, opening opportunities to analyze peptides that play critical roles in various diseases [121]. An MspA nanopore was functionalized with a copper (II) solution, enhancing its sensitivity. The unique conical geometry of MspA nanopores facilitates the translocation of small molecules, making copper (II)-modified MspA more sensitive than α-HL nanopores [122]. This modified nanopore was tested with an unnatural amino acid and 10 peptides, including those associated with neurological disorders and cancer. The method enables single-molecule analysis of peptides, allowing amino acids to be detected one by one and the peptide sequence to be deduced [122,123].

Beyond fundamental studies, analytical strategies involving biological nanopores have also shown promise in the detection of clinical biomarkers, particularly when tailored for selective peptide identification in complex biofluids. One such analytical approach has demonstrated the ability of α-HL biological nanopores to detect disease-specific peptides in patient samples, such as urine from individuals with ovarian cancer. Currently, routine ovarian cancer screening involves imaging (e.g., intravaginal ultrasound and MRI) or biomarker detection (e.g., CA-125 tumor antigen), but definitive diagnosis requires surgical intervention, which can be burdensome for patients. Identifying urinary peptides associated with ovarian cancer offers a less invasive solution. Using a system with an α-HL nanopore and two microwells, nanopore analysis facilitated peptide identification. In this system, one pipette introduces gold particles that capture cysteine-containing peptides, while the other introduces the peptides for analysis [124,125]. Shorter peptides, with fewer than 10 amino acids, showed faster interactions, while peptides with more than 10 amino acids allowed for extended interrogation times, thanks to the gold nanoparticles. This approach successfully identified cysteine-containing peptides from LRG-1 (Leucine-Rich Alpha-2-Glycoprotein 1), a protein biomarker specific to ovarian cancer that is present in the urine of affected women [126].

Identifying proteins directly from body fluids is ideal in the context of developing portable biosensors. Straathof et al. [57] propose a single nanopore sensor capable of identifying target proteins directly from blood, without pre-processing, by combining an entropic barrier with multivalent chemical recognition. The authors utilize the YaxAΔ40B variant [58], a decamer to which they attach a flexible polypeptide linker (approximately 70 amino acids) terminated with the StrepII peptide to the cis side [127]. These tails spontaneously collapse into a “mesh” that restricts the pore diameter and rejects nonspecific molecules. The system functions under conditions that preserve the native protein structure and minimize ion noise. It has been demonstrated that under the influence of an electric field, streptavidin can traverse the barrier due to the sequential binding of each of its four sites to the streptavidin tags on the chains. Once ensnared, the molecule becomes entrapped within the pore for several minutes, resulting in the generation of a current signal. The platform demonstrates the capacity to detect streptavidin at 1 nM even in 25% defibrinated blood, thereby exhibiting nanomolar sensitivity within a highly complex matrix [57].

The performance of the ONT method in terms of read length was the highest, processing proteins of approximately 300 amino acids using a ClpX motor molecule [116]. Repeating the read length process resulted in an accuracy of 99% [128]. In contrast, classical nanopores such as functionalized MspA [129] or α-HL [108] have been demonstrated to sequence only short peptides, typically tens of amino acids in length. The study and enhancement of this technique are more straightforward when conducted on single nanopore systems, which justifies their prevalence in fundamental research.

##### Protein Analysis with Solid-State Nanopore

Solid-state silicon nitride nanopores were explored, as solid nanopores are often more durable and resistant to varying conditions, with customizable sizes and shapes. Initial attempts at unfolding proteins for translocation through solid nanopores involved terminal denaturation or the application of high voltage. However, these physical methods often led to degradation of the protein structure [129,130,131,132,133,134,135]. Chemical denaturation emerged as a more effective approach. SDS (sodium dodecyl sulfate) was tested as a denaturing agent in solid-state nanopores, and it successfully disrupted the protein’s spatial structure, enabling translocation through the nanopore. After SDS treatment, the protein acquired a strong negative charge, facilitating translocation through electroosmotic flow due to the high surface charge of the denatured protein [130,136,137]. Electroosmosis, a phenomenon caused by the movement of counterions accumulated under the action of an applied electric field, contributes significantly to the capture of molecules in nanopores, independent of their charge. This flow mechanism allows the transport of molecules against their electrophoretic mobility direction [138,139,140].

Bovine serum albumin (BSA) was analyzed under varying pH conditions using a silicon nitride nanopore. This nanopore, embedded within a membrane, separates two equal-volume compartments connected by an electrophysiological solution. When an external voltage is applied, a stable baseline current is observed with the open nanopore. Upon introducing protein molecules, they are captured by the electric field and drawn through the nanopore. As each protein translocates through the pore, characteristic changes in current amplitude are recorded over time [141].

Given that this technique is intended for use at the point of care, it is important to simplify the sample preparation steps. The exploration of the potential of SiNx solid-state nanopores in the analysis of proteins at the monomolecular level directly from cell extracts has been successful [142]. The method was engineered by fusing the LOV2 model protein with a conductive protein (NEPD) expressed in 293T cells, then the cytoplasmic content of these individual cells was extracted using nanopipettes without lysing the cells. Subsequently, the contents of a single cell were transferred to the cis chamber of the fluidic cell containing the SiNX nanopore. The measurements obtained demonstrated significant disparities between the NEPD protein and the fused NEPD-LOV2 protein. These results provide validation for the capacity of nanopores to detect proteins directly from cells, as well as their ability to analyze molecular interactions in native biological environments.

##### Protein Analysis with Hybrid Nanopores

While Oxford Nanopore Technologies has made substantial advances, researchers continue to explore optimized variants specifically for protein analysis [143]. Solid-state nanopores are the most straightforward to modify physically; however, they are challenging to reproduce with consistent aperture sizes. Biological nanopores offer greater reproducibility and possess a uniform surface electric charge, but their lipid bilayer, which provides structural support, lacks durability under external force [144]. In theory, a hybrid nanopore that combines the advantages of both solid-state and biological nanopores could provide an ideal solution. Efforts have been made to create such a hybrid without a lipid bilayer by integrating helical molecules into silicon nitride (SiNx) nanopores [89,145]. This hybrid design allows for highly specific amino acid detection, as each amino acid generates a distinct ionic current blockade pattern. Uniquely, the method can also differentiate between amino acid enantiomers (e.g., L-Glu and D-Glu) based on their specific current signatures [146].

**Figure 3:**
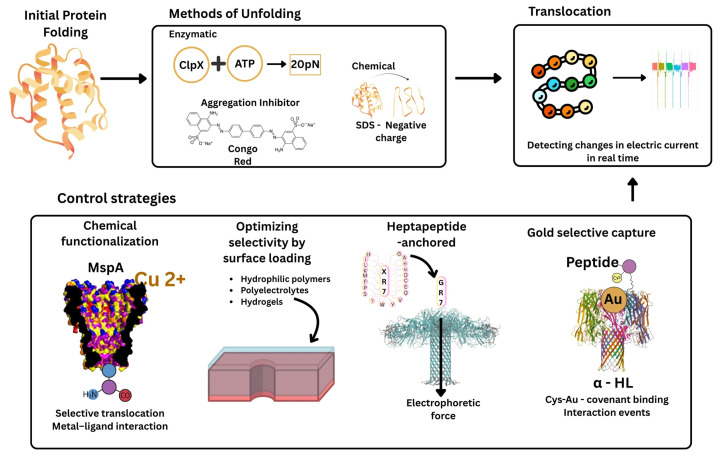
Schematic representation of protein translocation through nanopores. Unfolding of the native protein, accomplished either enzymatically (ClpX + ATP) or chemically (SDS, Congo Red). Translocation of the protein chain through the nanopore, generating real-time detectable ionic current blocks. Translocation control strategies: chemical pore functionalization (e.g., Cu^2+^-MspA), selectivity modification by surface charges, translocation slowing by heptapeptide anchoring, and selective trapping of cysteine-containing peptides by interactions with gold nanoparticles (α-HL) [110,122,126].

To overcome the current barriers, it is necessary to understand the progress and what has worked so far in the translocation process, below is a schematic representation (Figure 3) of protein translocation methods, including the unfolding and control strategies.

#### 4.2.3. Strategies for Nanopore Engineering and Optimization

Nanopore engineering is a central element for translocation strategies, each change in the system having a direct impact on system parameters (event duration and signal strength) [144]. One of the most widely used optimization methods for biological nanopores is mutagenesis, which enables site-specific modifications of the pore’s amino acid residues to improve performance. This involves the controlled replacement of some amino acids in the pore structure with others that modify the physico-chemical properties of the channel, such as electrical charge, constriction diameter, or wall hydrophobicity. For example, in the case of the aerolysin pore, mutations such as R220 or K238 [147] have been used to alter the electrostatic charge distribution, thereby achieving a significant increase in translocation duration and signal differentiation for peptides of similar compositions. In the MspA pore, the N91H mutation allows coordination of metal ions (Cu^2+^), generating a binding site with high specificity for amino acids [122].

Another nanopore engineering strategy involves the chemical functionalization of channel walls, with applicability to solid and hybrid nanopores. This approach consists of chemically modifying the pore inner surface by attaching functional groups, recognition molecules (e.g., aptamers and antibodies), polymers, or even inorganic layers to adjust molecular interactions, reducing noise, and increasing translocation fidelity. For example, salinization treatments with APTES introduce protonable amine groups that can invert or neutralize the surface charge, thereby influencing the attraction or repulsion of target molecules and controlling electroosmotic flow [148]. In the case of graphene [149] or MoS_2_ [150], the surfaces can be coated with hydroxyl or carboxyl groups, significantly altering channel hydrophobicity and slowing peptide translocation, allowing more detailed detection. These interactions induce distinct blocking signals, allowing clear discrimination between the analyte of interest and similar biomolecules. However, this method involves higher technological complexity, requiring stringent conditions for functional layer replication and fine calibration of translocation parameters.

Another essential tuning mechanism of the nanopore technique is the control of environmental conditions, in particular, pH, applied voltage, and ionic strength [85,109,130,151]. These variables influence the electrostatic properties of the channel and thus the translocation dynamics. Adjusting the pH changes the protonation state of ionizable groups (e.g., amino, carboxyl, and histidine) on the walls of both biological and solid-state nanopores, leading to variations in the net charge and surface potential that influence transport processes [152,153]. Also, the applied voltage plays a crucial role in forcing molecules to cross the pore—a higher voltage accelerates translocation but may compromise temporal resolution [154,155], whereas a moderate voltage ensures an optimal ratio between frequency and duration of events. The ionic strength of the solution, regulated by the concentration of salts (e.g., KCl), influences the shielding of electrostatic charges, which affects both the signal shape and the nonspecific interactions between the pore and analyte [156].

### 4.3. Analysis of Protein–Protein and Protein–Drug Interactions

Protein–protein interactions are fundamental to understanding cellular mechanisms, including molecular transport, cellular structure formation, and signal transduction [157]. In cancer, proteins regulate cell growth and division; in neurodegenerative diseases, protein modifications, such as amyloid-beta plaque formation and tau protein aggregation in Alzheimer’s disease, critically disrupt neuronal function [158]. Furthermore, the role of inhibitors and stabilizers in modulating protein–protein interactions supports the development of new drugs and therapies [159]. Studying these interactions is essential for understanding normal cell function and the pathogenesis of many diseases.

An innovative approach to observing and measuring such interactions involves the use of specially engineered biological nanopores, where the nanopore sensor is customized through genetic coding. For accurate quantification, protein–protein interactions must occur within an electrophysiological solution to detect changes in ion concentrations. In one study [160], interactions were observed by introducing two proteins into the cis side of the nanopore, with measurable changes in ion flow becoming more pronounced as the concentration of one protein was increased. The use of nanopore sensors to investigate protein–protein interactions was demonstrated with a nanopore constructed from t-FhuA, a truncated β-barrel protein from *Escherichia coli*. An inhibitory protein-ligand (Barstar, or Bs) was added to test protein–protein interactions. Transitions were detected upon the addition of Bs, with event duration increasing in line with Bs concentration, confirming a biomolecular binding interaction. This nanopore biosensor provides a sensitive, accurate, real-time method for measuring protein–protein interactions, with promising applications in clinical diagnostics and biomarker discovery [160].

Targeted drug discovery is integral to developing personalized medicine and studying protein–drug interactions with nanopores provides insights into mechanisms of action. Research into novel protein biomarkers is critically important and increasingly relevant in today’s scientific landscape. Additionally, investigating protein–drug interactions enhances drug efficacy and aids in the development of targeted therapies. Studying these interactions with label-free biological nanopores addresses some limitations of traditional methods, such as low sensitivity and restricted protein solubility. In a recent study [161], the pore-forming toxin YaxAB was used to examine the interaction between the B-cell lymphoma protein Bcl-xl and various ligands (ABT-737, A-1331852). Interactions between FKBP12 and the drug FK506 were also observed to validate the method. In both cases, specific events confirmed that nanopore analysis can effectively capture protein–drug interactions at the molecular level [124]. The unique pore shape formed by YaxAB toxin enables electroosmotic trapping of folded proteins and high-resolution monitoring of their interactions. Under specific conditions, the YaxAB pore exhibits strong cation selectivity, creating an electroosmotic flux that enhances protein trapping. Several folded proteins, including Bcl-xL, FKBP12, and MDM2, were successfully captured, and their interactions with drugs were monitored. The recorded events demonstrated the sensor’s capability to distinguish when proteins interact with a drug versus when they do not, marking an important step toward new drug discoveries [161].

Nanopore technology was also used to study protein-binding interactions relevant to cancer treatment. The p53 protein, essential in cancer prevention, activates genes that halt cell growth or trigger apoptosis, while MDM2 inhibits p53, preventing it from performing its tumor-suppressing functions. Using a solid-state silicon nitride (SiNx) nanopore, researchers investigated this interaction. Blocking MDM2 from binding to p53 is crucial in cancer therapy, as it allows p53 to remain active and destroy cancerous cells. MDM2 translocation through the nanopore was enabled by applying a negative voltage and adding Nutlin-3, an inhibitor of the MDM2-p53 interaction. The p53 protein exhibited varying conformations, reflected in the distribution of event durations, revealing important details about its interaction dynamics [162].

## 5. Signal Analysis and Bioinformatics Approach

In nanopore signal processing, various machine learning and artificial intelligence algorithms can be employed to improve data interpretation. Bioinformatics analysis is essential for nanopore experiments, as the signal is influenced by numerous factors (e.g., pore size, molecular structure, buffer composition, and temperature). Additionally, structural variations within the protein can alter the signal in unpredictable ways, reducing analytical accuracy. Given these complexities, standardized signal analysis methods are critical, especially when using nanopores for diagnostic purposes. However, despite the importance of signal interpretation, significantly fewer studies focus on this aspect compared to electronic setup optimization. Current research highlights some specific features of nanopore signal processing. When a molecule translocates through a nanopore, the detected signals’ amplitude is lower than the baseline signal observed when the pore is open. Therefore, signal analysis requires identifying this current drop to establish levels. The first step is determining the baseline value, which serves as a reference point for subsequent measurements specific to the target molecule. The next step involves identifying the maximum and minimum points to delineate signal levels, allowing for distinct clustering. Without identifying these points, adjacent levels may be grouped as a single cluster rather than distinct ones. Once these key points are identified, they are clustered using the DBSCAN algorithm [163] with a threshold adjusted according to the baseline value. The signal may contain multiple levels, and sharp signal changes are detected using the findchangept function, representing individual levels within events and defining unique regions that vary from event to event. To prevent over-segmentation, the algorithm assesses the average current difference between adjacent levels; if the difference is below a user-defined threshold, the levels are combined [164].

Bioinformatics plays a crucial role in automating protein analysis techniques, especially in classifying the 20 amino acids using machine learning. This process begins with data import, followed by the extraction of defining features to build predictive models. In one study, researchers trained an algorithm using 1000 events from each of the 20 amino acid types, focusing on features such as mean blockade, dwell time, and normalized signal density.

**Figure 4:**
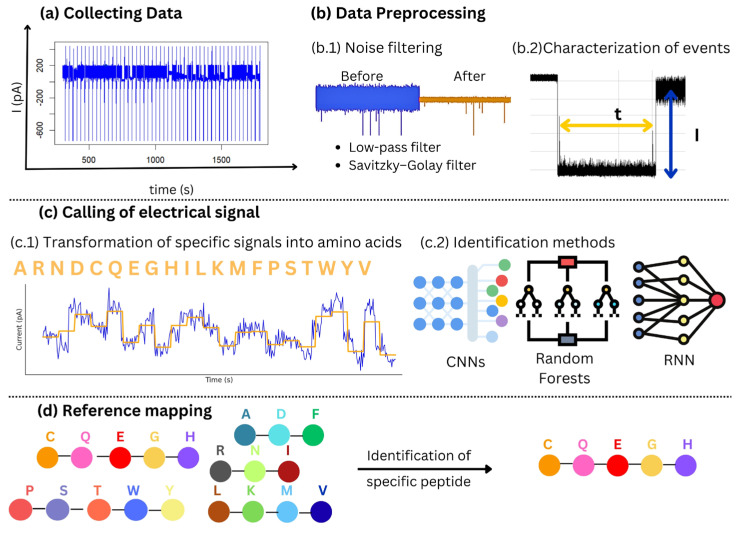
Schematic representation of signal processing. (**a**) Data collection involves recording the ionic current over time. (**b**) Preprocessing includes noise filtering (e.g., low-pass filter and Savitzky–Golay filter) and characterization of events according to duration (t) and amplitude (I). (**c**) Calling the electrical signal involves converting the signal into amino acid sequences (**c.1**) using machine learning methods such as convolutional neural networks (CNNs), random forests, or recurrent neural networks (RNNs) (**c.2**). (**d**) Reference mapping allows the signals obtained to be aligned with a database for the identification of specific peptides.

These parameters were analyzed with various machine learning models, including Random Forest (RF) [165], Neural Network (NNet) [166,167], and Classification And Regression Trees (CART) (Figure 4), which were then compared for accuracy and efficiency [168]. The algorithms developed for analyzing signals from nanopore translocation events are optimized for rapid, large-scale data processing to enable real-time analysis [144].

To address challenges such as overlapping current blockade signals during peptide translocation, advanced signal processing methods have been employed. Machine learning algorithms, including random forests (RF) and neural networks (NNet), were applied to improve amino acid discrimination. These approaches helped correlate average current blockades with amino acid volume, although deviations were observed for charged amino acids, indicating the need for more robust models [122,123], see.

Nano-Align is an algorithm designed for protein identification using nanospectra generated from the translocation of denatured, linearly charged proteins through sub-nanometer nanopores. The algorithm matches these nanospectra with entries in a protein database, providing *p*-values to indicate the statistical significance of each protein–nanospectra match. In the initial analysis step, Nano-Align processes raw data to extract nanospectra. Subsequent steps involve constructing a database of known proteins and using statistical assessments to evaluate similarity between the extracted spectra and database entries. The algorithm then reports *p*-values for each match, indicating the confidence level of protein identification [169,170].

Machine learning shows great promise for analyzing proteins via nanopore techniques. Classifiers such as Random Forest (RF) and Rotation Forest have been applied, with RF demonstrating up to 6% higher F-values compared to Rotation Forest, indicating superior performance. Protein data were binarized, and the test set included randomly combined data from various experiments. Ultimately, F-values reached up to 99.3% when distinguishing between proteins of similar sizes, supporting the feasibility of this approach [171].

The NanopreTERs application, specifically designed for nanopore-based protein analysis, integrates machine learning algorithms. It processes raw data files by applying noise reduction and signal normalization filters. The data are then restructured for compatibility with a Convolutional Neural Network (CNN) model and divided into training, validation, and test sets. The CNN model is trained on the training set, and its performance is evaluated on the validation set. RF was also tested on these datasets and demonstrated higher accuracy than CNN. However, CNN may be more suitable in experiments with an increased number of barcodes, where complex data patterns require deeper learning [172,173].

AutoStepfinder is an analysis tool designed to identify discrete steps in nanopore signals without requiring prior information. The algorithm partitions data into subsets, minimizing the discrepancy between observed and fitted values. AutoStepfinder, an extension of Stepfinder, evaluates the step-fitting spectrum (S-curve) within the data, allowing for component analysis where steps at various scales are separated and analyzed independently. This versatile algorithm can be applied across multiple experimental domains [174]. In the context of biomarker discovery, exploring how solid-state nanopores combined with machine learning can improve the identification accuracy of size-similar proteins is crucial. Four proteins of similar size were evaluated using amplifiers at 100 kHz and 10 MHz frequency bands. Significant differences emerged when machine learning was applied, with classification improved by grouping signals based on specific characteristics. This approach yielded F-values up to 88.7% and specificities up to 96.4%, demonstrating the method’s capability to distinguish between similar proteins [171].

## 6. Comparative Advantages over Traditional Methods

Nanopore technology demonstrates remarkable sensitivity advantages over conventional protein detection methods. Studies have shown detection limits reaching femtomolar to attomolar concentrations, significantly outperforming traditional ELISA assays [49,175]. For instance, nanopore-based enzyme-linked immunosorbent assays have achieved detection limits as low as 0.03 fg/mL for cancer biomarkers [175], representing orders of magnitude improvement over conventional approaches.

The single-molecule detection capability of nanopores enables direct quantification without the need for signal amplification steps required in traditional immunoassays [176]. This fundamental advantage allows for the detection of biomarkers present at extremely low concentrations, particularly relevant for early-stage disease detection where biomarker levels may be below the threshold of conventional assays [51].

Unlike traditional methods that require hours to days for completion, nanopore-based assays can provide results within minutes to hours [9,48]. The immunoprecipitation-coupled nanopore (IP-NP) assay demonstrates the ability to detect pathogen-derived peptides from serum samples with rapid turnaround times suitable for clinical decision-making [9]. This speed advantage is particularly crucial for point-of-care applications where immediate results can influence treatment decisions.

Also, traditional protein detection methods typically require fluorescent, enzymatic, or radioactive labels, which can interfere with protein function and require additional processing steps [177]. Nanopore technology enables direct, label-free detection by measuring intrinsic electrical signatures of proteins as they interact with or traverse the nanopore [62,177]. This approach eliminates labeling artifacts and reduces assay complexity while maintaining high specificity.

Regarding the multiplexing and high-throughput potential, nanopore platforms demonstrate superior multiplexing capabilities compared to traditional single-analyte assays. Studies have shown simultaneous detection of multiple biomarkers using different nanopore signatures or DNA-assisted detection strategies [50,178]. The ability to detect multiple biomarkers in a single assay reduces sample volume requirements and analysis time while providing comprehensive diagnostic information.

## 7. Challenges and Opportunities

Nanopore technology offers significant potential for molecular analysis and is paving the way for advancements in personalized medicine and molecular discoveries. By analyzing proteins one amino acid at a time and capturing interactions between proteins and other molecules within nanopores, this technique overcomes some of the limitations of traditional methods like mass spectrometry and Edman degradation, providing label-free detection with high sensitivity. The primary challenges in nanopore-based protein analysis include controlling the capture and translocation of molecules. This control depends on factors such as nanopore sensitivity and the charge distribution along the peptide. Unlike DNA, which has a uniform charge distribution, proteins exhibit uneven charge along their amino acid sequence. This results in variable electroosmotic forces that can cause uncontrolled movement through nanopores. Additionally, proteins often experience non-specific interactions with charged nanopore walls, as regions with different charges along the protein sequence interact variably with the nanopore interior, further complicating translocation and signal interpretation. The complex three-dimensional structure of proteins (e.g., alpha-helices and beta-sheets) also poses challenges, as it can hinder entry into the nanopore and cause blockages during translocation. Protein unfolding typically requires additional factors, such as applied forces or specific chemical conditions, that maintain the integrity of the protein’s chemical structure [179]. Another limiting factor in nanopore experiments is the signal-to-noise ratio [180]. Different types of noise, including flicker, white, dielectric, and capacitive noise, affect signal quality, and targeted adjustments can enhance method accuracy. Such adjustments might involve increasing the number of charge carriers or optimizing signal amplification to clearly distinguish translocation events. These adjustments vary based on the specific molecule being analyzed and the type of nanopore used (biological, synthetic, or hybrid) [181,182,183]. Future directions focus on developing nanopores that remain stable under diverse experimental conditions by combining biological components with solid-state materials to enhance stability and precision [184,185,186]. Additionally, the system’s chemistry is crucial, as translocation can be facilitated by motor molecules, such as enzymes, exopeptidases, or other guiding molecules, that help draw the molecule of interest through the pore [179].

## 8. Conclusions

Nanopores represent a significant innovation in DNA sequencing, and the studies discussed here show that nanopore technology holds great promise for protein analysis as well. This opens new avenues in personalized medicine and the discovery of biomarkers and mechanisms that regulate various biological processes. Nanopore analysis enables real-time, single-molecule monitoring, overcoming limitations of traditional methods like mass spectrometry and Edman degradation. Despite the advanced development of nanopore technology, several aspects need to be addressed, particularly in controlling molecule capture and translocation. The non-uniform charge distribution along the amino acid sequence and the complex three-dimensional structure of proteins complicates optimal translocation through nanopores. Efforts to address these challenges include developing hybrid nanopores that offer enhanced stability and precision and incorporating motor molecules to unfold proteins, enabling amino acid-by-amino acid sequencing. These advancements promise to improve analytical capabilities, enabling detailed protein characterization, including insights into protein–protein and protein–drug interactions. Due to the complexity of nanopore signals—which are influenced by factors such as nanopore properties, protein structure, analysis medium, and experimental conditions—accurate data interpretation requires sophisticated algorithms and advanced bioinformatics expertise. Applying this analytical technique to study proteins and their interactions offers tremendous potential for biomedical research and therapeutic development. Although challenges remain, current research indicates that nanopores could play a transformative role in clinical practice, revolutionizing our understanding and treatment of diseases at the molecular level and driving the advancement of personalized medicine.

## Figures and Tables

**Table 1:** Standard techniques in peptide and protein quantification and/or detection.

Method	Principle of the Method	Advantages	Disadvantages	Refs.
X-ray crystallography	Using a mobile and stationary phase system separate molecules according to their physico-chemical properties	Allows precise protein separations	The process can be complex and time-consuming	[30,31,32,33]
Colorimetric tests	The change in color of a solution when a specific molecule is present, measured using a spectrophotometer	Rapid and easy to use	Requires specific reagents for each target molecule	[34,35]
ELISA (Enzyme-Linked Immunosorbent Assay) and Western blot	Specific recognition between an antibody and an antigen, followed by detection of the signal generated by an enzyme bound to a secondary antibody	Speed, ease of use, and high sensitivity	Need for specific antibodies	[36,37,38]
Mass spectrometry	Involves analysis of the mass of protein molecules	Precise identification, analysis of post-translational modifications	Complex interpretation, limitations related to protein size	[39,40]
Electrophoresis	Separation of proteins according to their electrical charge and size	Efficient separation, versatility	Lower resolution, depending on the references used for sizing	[41,42]
Nuclear magnetic resonance (NMR)	Uses the magnetic properties of atomic nuclei to obtain structural and dynamic information about molecules	Atomic-level resolution, ability to study dynamics	Low sensitivity, data interpretation complexity, size limitations	[43,44]
Nanopores	Proteins can change the electrical conductivity of the system, which can be measured and interpreted	Single-molecule analysis, real-time monitoring	Improving sensitivity, complex interpretation	[45,46]

## Abbreviations

The following abbreviations are used in this manuscript:

ONT	Oxford Nanopore Technologies
MRI	Magnetic Resonance Imaging
CA-125	Cancer Antigen 125
ELISA	Enzyme-Linked Immunosorbent Assay
SDS	Sodium Dodecyl Sulfate
HD	Huntington’s Disease
CRP	C-Reactive Protein
PSA	Prostate-Specific Antigen
NMR	Nuclear Magnetic Resonance
RF	Random Forest
NNet	Neural Network
MspA	Mycobacterium smegmatis porin A
ATP	Adenosine Triphosphate
α-HL	Alpha-hemolysin
ClyA	Cytolysin A
OmpG	Outer membrane protein G
DNA	Deoxyribonucleic acid
CR	Congo Red
LRG-1	Leucine-Rich alpha-2-Glycoprotein 1
NNet	Neural Networks
PTMs	Post-Translational Modifications
CART	Classification And Regression Trees
RNNs	Recurrent Neural Networks
